# Identification of metabolic syndrome using phenotypes consisting of triglyceride levels with anthropometric indices in Korean adults

**DOI:** 10.1186/s12902-020-0510-0

**Published:** 2020-02-27

**Authors:** Bum Ju Lee, Jong Yeol Kim

**Affiliations:** Future Medicine Division, Korea Institute of Oriental Medicine, 1672 Yuseongdae-ro, Yuseong-gu, Deajeon, 305-811 Republic of Korea

**Keywords:** Metabolic syndrome, Anthropometry, Hypertriglyceridemic waist phenotype, Identification, Indicator, Korean population, Public health

## Abstract

**Background:**

The metabolic syndrome (MetS) has shown strong associations with the hypertriglyceridemic waist (HW) phenotype. The best anthropometric indicator of MetS remains controversial. Furthermore, no studies have investigated alternative indices that could replace waist circumference in the HW phenotype. The objectives of this study were to find the best indicator of MetS among anthropometric indices and to examine the predictive power of phenotypes consisting of triglyceride levels with anthropometric indices.

**Methods:**

A total of 12,025 subjects participated in this retrospective cross-sectional study. All subjects were recruited between November 2016 and August 2007 from hospitals in 28 urban and rural regions in South Korea. The data analyzed in this study were obtained from the Korean Health and Genome Epidemiology Study database and the Korea Institute of Oriental Medicine.

**Results:**

The proportion of patients with MetS ranged from 9 to 57% according to age and gender groups. Waist circumference (WC) was best indicator of MetS in men of all age groups. However, in women aged 40–49 years, the waist-to-height ratio (WHtR) was the best indicator of MetS. Rib circumference and chest circumference were the strongest indicators in women aged 50–59 years and 70–79 years, respectively. The combination of WC and triglyceride (TG) was the best indicator of MetS in men and women overall. However, interestingly, the best indicator was TG + WHtR in women aged 40–49 years and TG + forehead-to-waist ratio in women aged 70–79 years.

**Conclusions:**

The best indicator of MetS in terms of individual anthropometric indices and the various phenotypes combining a single anthropometric index with TG may differ subtly according to age group in women, but not in men. Our findings provide insight into a simple and inexpensive method that could be used to identify MetS in initial health screening efforts in epidemiology and public health.

## Background

The metabolic syndrome (MetS) is a very common metabolic disorder and has become one of the most important public health problems worldwide [1, 2]. The MetS consists of abdominal obesity, hypertriglyceridemia, hypo-high-density lipoprotein (HDL) cholesterolemia, hyperglycemia, and hypertension and directly facilitates the development of type 2 diabetes mellitus and atherosclerotic cardiovascular disease; furthermore, MetS increases mortality due to cardiovascular disease [1, 3, 4]. The prevalence of MetS is very high in middle and old age and increases with obesity [5, 6]. Although many studies have assessed the associations between MetS and anthropometric indices, the index that best predicts MetS remains controversial, despite the fact that waist circumference (WaistC) is one of the five components of MetS. WaistC has been shown to be the best indicator of MetS in Iranian adults [7], Chinese adults [8], and Qatari adults [9], whereas the waist-to-height ratio (WHtR) was found to be the best indicator of MetS in Mexican children [10], Japanese adults [11], Chinese adults [12], and an Italian population [13].

Recent studies have suggested using the hypertriglyceridemic waist (HW) phenotype as an alternative indicator of chronic diseases such as MetS [14], metabolic abnormalities [15], and diabetes [16] because of its simple and cost-effective measurement. Compared to obese women with either MetS or the HW phenotype, those with both MetS and the HW phenotype experience aggravated cardiometabolic risks and insulin resistance [17]. The HW phenotype has been associated with low education level, age, and a sedentary job [14]. This phenotype generally consists of triglyceride (TG) and WaistC and is diagnosed when values of TG and WaistC exceed a specific threshold. Many studies to date have suggested the importance of the HW phenotype in diagnosing MetS or have reported an association between HW and MetS [14, 15, 18–22]. However, these previous studies did not examine other circumference or ratio indices that could replace WaistC in the HW phenotype to identify MetS.

The objectives of the present study were to determine the best indicator of MetS among various anthropometric indices in Korean adults and to examine the predictive power of various phenotypes consisting of combinations of individual anthropometric indices and TG levels to identify MetS. To our knowledge, this is the first report to analyze the association of individual anthropometric indices with MetS and the predictive power of various phenotypes using combinations of individual anthropometric indices and TG to diagnose MetS in Korean adults.

## Methods

### Study population and data source

A total of 12,025 subjects (4936 men and 7089 women aged 30–79 years) participated in this retrospective cross-sectional study. All subjects were recruited between November 2016 and August 2007 from hospitals in 28 urban and rural regions including Anseong, Ansan, and other cities. The data analyzed in the present study were obtained from the Korean Health and Genome Epidemiology Study (KHGES) database and the Korea Institute of Oriental Medicine (KIOM). All subjects participated in the study voluntarily. Written informed consent was obtained from all participants. The KIOM Institutional Review Board (IRB) approved this study (No. I-1210/002/002–02), and this study was performed in accordance with the relevant guidelines and regulations by the IRB of the KIOM, the Ajou University Hospital (AJIRB-MED-SUR-12-377), the Korea University Ansan Hospital (AS10153), and each TKM hospitals. This study was conducted according to the standards of the International Committee on Harmonization on Good Clinical Practice and the revised version of the Declaration of Helsinki.

To select the sample, the following inclusion criteria were applied: subjects who 1) provided written informed consent; 2) were aged 30–79 years; and 3) were Koreans residing in the Republic of Korea. The exclusion criteria were as follows: 1) subjects missing anthropometric index, blood pressure, or blood parameter information; 2) subjects missing basic characteristics such as age, education, region, or gender; and 3) subjects with other missing data.

### Anthropometry and measurement

Participants’ blood parameters including fasting plasma glucose (FPG) and TG levels were measured to diagnose MetS and to determine HW phenotype. All participants were asked to fast for at least 8 h, and blood samples were subsequently drawn to analyze blood parameters (ADVIA 1800, Siemens, USA) [23].

This study extracted and used more detailed and various anthropometric indices than many previous studies. The specific area of the body that is measured is crucial to identifying obesity-related diseases because subtle differences in the measured positions have been related to the power to identify risk of health outcomes [24, 25]. The various anthropometric indices used in this study were obtained by well-trained physicians or observers based on standardized protocols. Subjects’ weight and height were measured to the nearest 0.1 cm and 0.1 kg, respectively (LG-150; G Tech International Co., Ltd., Uijeongbu, Republic of Korea). Eight circumferences, namely, the forehead (ForeheadC), neck (NeckC), axilla (AxillaryC), chest (ChestC), rib (RibC), waist (WaistC), pelvis (PelvicC), and hip (HipC), were measured in the corresponding locations with subjects wearing lightweight clothing and no shoes. ForeheadC was NeckC were measured at the levels of the glabella and occiput of the head and at the levels of the thyroid cartilage and cricoid cartilage, respectively. AxillaryC was gauged at the levels of the left and right axillae. ChestC was gauged at the levels of the left and right nipples, and RibC was measured at the levels of the left and right 7th and 8th prominences of the costochondral junction. WaistC and PelvicC were gauged at the level of the umbilicus and at the levels of the left and right anterior superior iliac spines, respectively. HipC was gauged at the level of the upper edge of the pubis [15, 23, 25]. Based on these circumferences, we computed the ratios between indices that are commonly used in medicine, anthropometry, and epidemiology. Finally, we extracted several ratios including WHR, WHtR, forehead-to-waist ratio (Forehead_Waist), forehead-to-rib ratio (Forehead_Rib), and body mass index (BMI). More details regarding the measurement positions, ratio indices, and descriptions of variables have been provided in previous studies [23, 25, 26]. The basic characteristics and a brief description of all the variables used in this study are described in Supplementary Table 1, and baseline characteristics between normal and MetS groups in men and women are described in Supplementary Table 2.

### Definition

To diagnose MetS, we considered the recommendations of the National Cholesterol Education Program Adult Treatment Panel III (NCEP ATP III) [27]. Based on the criteria outlined by the NCEP ATP III, subjects having 3 or more of the following criteria were diagnosed with MetS: 1) blood pressure: ≥130/85 mmHg; 2) fasting glucose: ≥110 mg/dL (≥6.1 mmol/L); 3) triglycerides: ≥150 mg/dL (1.69 mmol/L); 4) HDL cholesterol: < 40 mg/dL (1.04 mmol/L) in men and < 50 mg/dL (1.29 mmol/L) in women; and 5) abdominal obesity: WaistC > 90 cm in men and > 80 cm in women [3, 27, 28]. Several previous studies and the World Health Organization (WHO) recommendations have suggested that WaistC values > 88 cm in women and > 102 cm in men are not suitable for determining abdominal obesity in Asian populations [29–31]. Therefore, in this study, we used modified WaistC criteria (> 90 cm in men and > 80 cm in women) according to the recommendations of previous studies and the WHO [29–31].

To determine HW phenotype, we considered the definition of the HW phenotype reported in recent studies [23, 32–35].The HW phenotype was defined as follows: TG ≥133 mg/dl (1.5 mmol/L) and WaistC ≥85 cm in women and TG ≥177 mg/dl (2.0 mmol/L) and WaistC ≥90 cm in men. Therefore, men with TG < 177 mg/dl (2.0 mmol/L) and/or WaistC < 90 cm and women with TG < 133 mg/dl (1.5 mmol/L) and/or WaistC < 85 cm were considered normal participants.

### Statistical analysis

The statistical analyses to calculate associations and predictive power were conducted with SPSS 23 for Windows (SPSS Inc., Chicago, IL, USA) and the Waikato Environment for Knowledge Analysis (WEKA) data mining tool [36]. In both the crude analysis and the analysis adjusted for age, region, and education, binary logistic regression was performed to examine the significant differences between the normal group and the MetS group after transforming all data in a standardized manner. To compare the predictive power of individual variables and the various phenotypes consisting of individual anthropometric indices and TG, we considered the main criterion as the area under the receiver operating characteristic curve (AUC) using 10-fold cross-validation to validate the model; the AUC value was selected because it is typically used to evaluate the predictive power of indicators and classifications in medicine and biology research. Additionally, we analyzed the predictive power of the combined measurements using the actual value of one anthropometric index and TG values to verify whether the combination of WaistC and TG, the components of the HW phenotype, was the best indicator of MetS.

To obtain more details according to age, our data were divided into 10 sub-groups by age and gender. Specifically, the groups consisted of men aged 30–39 years (M-30-39 group), men aged 40–49 years (M-40-49 group), men aged 50–59 years (M-50-59 group), men aged 60–69 years (M-60-69 group), men aged 70–79 years (M-70-79 group), women aged 30–39 years (W-30-39 group), women aged 40–49 years (W-40-49 group), women aged 50–59 years (W-50-59 group), women aged 60–69 years (W-60-69 group), and women aged 70–79 years (W-70-79 group).

## Results

**Fig. 1:**
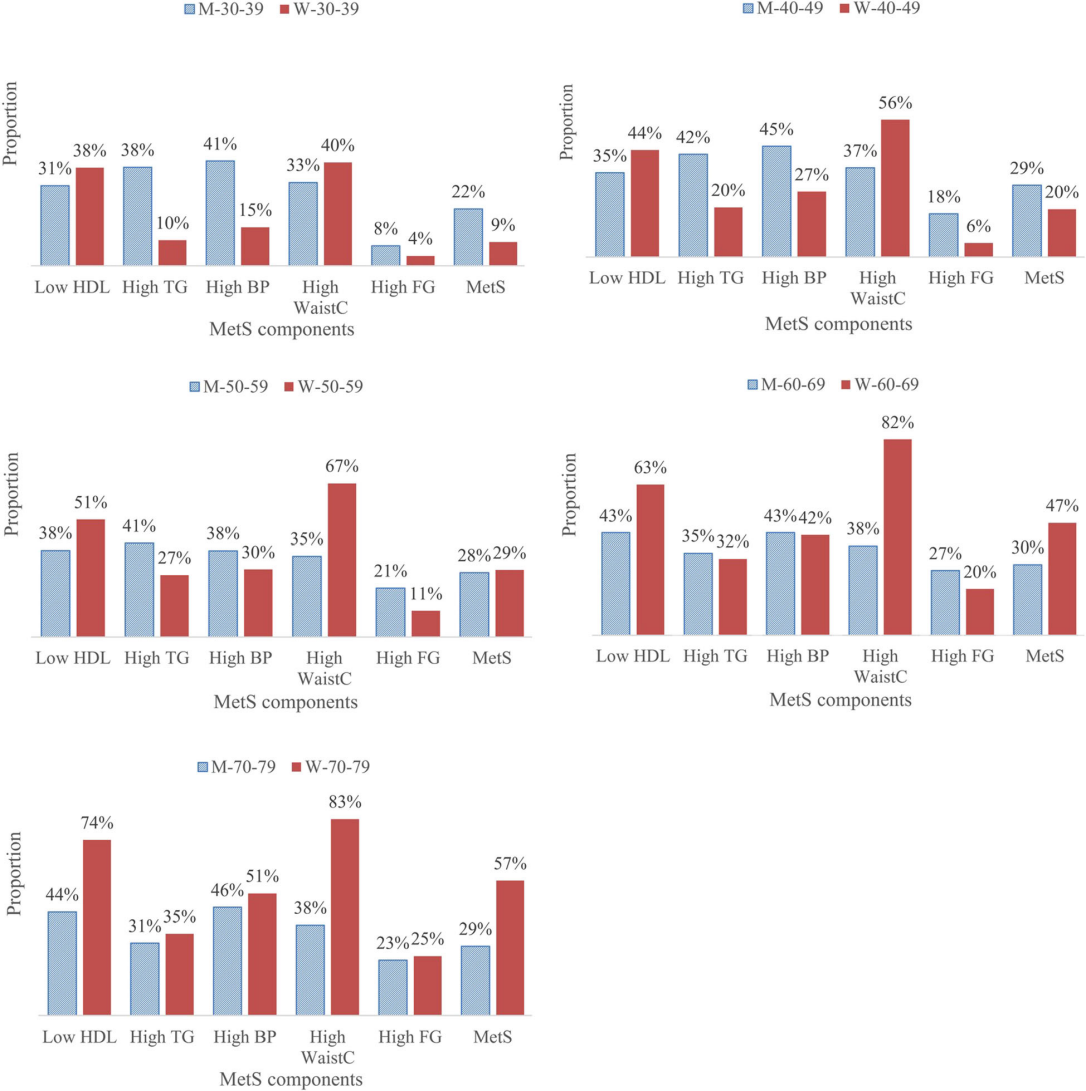
The proportion of patients with the metabolic syndrome and proportion of subjects with each MetS component according to age and gender groups. MetS, metabolic syndrome; M-30-39, men aged 30–39 years; M-40-49, men aged 40–49 years; M-50-59, men aged 50–59 years; M-60-69, men aged 60–69 years; M-70-79, men aged 70–79 years; W-30-39, women aged 30–39 years; W-40-49, women aged 40–49 years; W-50-59, women aged 50–59 years; W-60-69, women aged 60–69 years; W-70-79, women aged 70–79 years

The proportion of patients with MetS ranged from 9 to 57% according to age and gender groups (Fig. 1). The highest proportions of MetS were in women aged 70–79 years (W-70-79), at 57%, and in men aged 40–79 years, at 28–30%. Of the five MetS components, high WaistC was the most prevalent in all groups of women except for those aged 30–39 years. Regarding the groups of men, the proportions of high TG and high blood pressure (BP) were the highest in men aged 30–39 years (38 and 41%, respectively) and those aged 40–49 years (42 and 45%, respectively). However, the proportions of low HDL and high BP were the highest in men aged 60–69 years (43 and 43%, respectively) and those aged 70–79 years (44 and 46%, respectively). The proportion of MetS was much lower in middle-aged women than in men of the same age, but the proportion of MetS was much higher in older women than in men of the same age.

### Association of MetS with individual anthropometric indices

Tables 1, 2, 3, 4 and 5 list the associations between MetS and anthropometric indices and the power of the individual indices and the combinations of TG and these indices to identify MetS according to each age group of men and women.

**Table 1:** Analysis of the association between MetS and anthropometric indices and the predictive power for MetS in men and women aged 30–39 years

Index	M-30-39						W-30-39					
	OR	*p*	Adj. OR	Adj. p	AUC-1	AUC-2	OR	*p*	Adj. OR	Adj. p	AUC-1	AUC-2
Height	1.38 (1.09–1.74)	.008	1.49 (1.16–1.92)	.002	0.586	0.862	0.97 (0.76–1.23)	.793	1.07 (0.84–1.38)	.578	0.471	0.873
Weight	2.58 (1.97–3.38)	< .001	2.64 (1.99–3.51)	< .001	0.769	0.885	3.35 (2.59–4.31)	< .001	3.87 (2.87–5.21)	< .001	0.851	0.926
BMI	2.57 (1.94–3.4)	< .001	2.52 (1.89–3.37)	< .001	0.770	0.882	3.53 (2.73–4.55)	< .001	4.01 (2.97–5.41)	< .001	0.861	0.931
ForeheadC	1.88 (1.45–2.43)	< .001	1.96 (1.48–2.6)	< .001	0.661	0.874	2.01 (1.53–2.64)	< .001	2.62 (1.89–3.62)	< .001	0.641	0.876
NeckC	2.75 (2.06–3.67)	< .001	2.7 (2–3.64)	< .001	0.749	0.88	3.43 (2.62–4.49)	< .001	3.72 (2.74–5.04)	< .001	0.808	0.91
AxillaryC	2.86 (2.15–3.8)	< .001	3.04 (2.23–4.16)	< .001	0.761	0.883	4.29 (3.21–5.74)	< .001	4.81 (3.45–6.7)	< .001	0.869	0.929
ChestC	2.83 (2.14–3.74)	< .001	3.02 (2.22–4.11)	< .001	0.764	0.883	3.76 (2.86–4.94)	< .001	4.76 (3.42–6.63)	< .001	0.857	0.923
RibC	3.3 (2.4–4.55)	< .001	3.24 (2.32–4.53)	< .001	0.808	0.887	3.81 (2.9–4.99)	< .001	4.33 (3.17–5.93)	< .001	0.879	0.931
WaistC	3.6 (2.59–5)	< .001	3.85 (2.69–5.5)	< .001	0.825	0.903	4.47 (3.3–6.04)	< .001	5.24 (3.66–7.52)	< .001	0.883	0.938
PelvicC	2.35 (1.79–3.1)	< .001	2.62 (1.92–3.57)	< .001	0.739	0.877	3.44 (2.63–4.5)	< .001	4.06 (2.95–5.57)	< .001	0.814	0.919
HipC	2.25 (1.73–2.93)	< .001	2.68 (1.98–3.63)	< .001	0.725	0.872	2.78 (2.17–3.56)	< .001	3.31 (2.47–4.44)	< .001	0.778	0.911
Waist_Hip	2.99 (2.2–4.05)	< .001	3.02 (2.16–4.21)	< .001	0.771	0.891	3.06 (2.31–4.06)	< .001	3.02 (2.21–4.11)	< .001	0.819	0.915
Waist_Pelvic	2.58 (1.92–3.45)	< .001	2.7 (1.97–3.7)	< .001	0.740	0.886	2.38 (1.8–3.14)	< .001	2.25 (1.67–3.03)	< .001	0.749	0.9
Forehead_Waist	0.28 (0.2–0.39)	< .001	0.26 (0.18–0.37)	< .001	0.795	0.892	0.21 (0.15–0.29)	< .001	0.18 (0.12–0.27)	< .001	0.859	0.935
Forehead_Rib	0.32 (0.23–0.45)	< .001	0.32 (0.23–0.45)	< .001	0.773	0.875	0.25 (0.19–0.34)	< .001	0.23 (0.16–0.32)	< .001	0.854	0.932
Forehead_Chest	0.39 (0.3–0.52)	< .001	0.37 (0.27–0.5)	< .001	0.727	0.872	0.28 (0.21–0.37)	< .001	0.22 (0.16–0.32)	< .001	0.821	0.921
WHtR	3.31 (2.4–4.58)	< .001	3.38 (2.4–4.75)	< .001	0.809	0.891	4.15 (3.11–5.52)	< .001	4.54 (3.27–6.32)	< .001	0.879	0.935
TG	5.82 (3.76–8.99)	< .001	5.68 (3.63–8.89)	< .001	0.867	–	4.63 (3.44–6.22)	< .001	5.21 (3.71–7.32)	< .001	0.876	–
HW phenotype	0.02 (0.01–0.04)	< .001	0.02 (0.01–0.04)	< .001	–	–	0.01 (0.01–0.03)	< .001	0.01 (0.01–0.02)	< .001	–	–

The results were obtained by binary logistic regression. *M-30-39* men aged 30–39 years, *W-30-39* women aged 30–39 years, *Adj. p and OR* adjustment for age, region, and education, *OR* odds ratio, *AUC-1* AUC value of each index used to identify MetS, *AUC-2* AUC value of phenotypes combining TG + one anthropometric index to identify MetS

**Table 2:** Analysis of the association between MetS and anthropometric indices and the predictive power for MetS in men and women aged 40–49 years

Index	M-40-49						W-40-49					
	OR	*p*	Adj. OR	Adj. p	AUC-1	AUC-2	OR	*p*	Adj. OR	Adj. p	AUC-1	AUC-2
Height	1.14 (0.97–1.33)	.109	1.12 (0.94–1.33)	.193	0.537	0.825	0.87 (0.76–0.99)	.041	0.95 (0.82–1.09)	.439	0.53	0.847
Weight	3.15 (2.54–3.9)	< .001	3.51 (2.77–4.44)	< .001	0.766	0.862	2.36 (2.04–2.74)	< .001	2.4 (2.06–2.8)	< .001	0.741	0.888
BMI	3.47 (2.78–4.34)	< .001	3.82 (3–4.87)	< .001	0.777	0.867	2.79 (2.38–3.27)	< .001	2.78 (2.36–3.27)	< .001	0.777	0.896
ForeheadC	1.64 (1.39–1.94)	< .001	1.75 (1.45–2.11)	< .001	0.621	0.834	1.15 (1.01–1.31)	.043	1.27 (1.09–1.44)	.002	0.538	0.852
NeckC	3.42 (2.74–4.28)	< .001	4.16 (3.21–5.39)	< .001	0.771	0.866	2.26 (1.94–2.64)	< .001	2.39 (2.03–2.82)	< .001	0.717	0.879
AxillaryC	2.99 (2.42–3.69)	< .001	3.17 (2.52–3.99)	< .001	0.765	0.861	2.93 (2.48–3.46)	< .001	2.95 (2.47–3.52)	< .001	0.775	0.888
ChestC	3.13 (2.53–3.88)	< .001	3.2 (2.55–4.02)	< .001	0.773	0.865	3 (2.54–3.55)	< .001	3.02 (2.53–3.61)	< .001	0.783	0.895
RibC	4.06 (3.21–5.15)	< .001	4.36 (3.37–5.64)	< .001	0.806	0.878	2.73 (2.33–3.19)	< .001	2.68 (2.27–3.17)	< .001	0.776	0.891
WaistC	4.66 (3.6–6.02)	< .001	4.95 (3.75–6.52)	< .001	0.824	0.889	2.89 (2.45–3.41)	< .001	2.89 (2.43–3.44)	< .001	0.78	0.895
PelvicC	2.96 (2.4–3.65)	< .001	3.41 (2.7–4.31)	< .001	0.754	0.859	2.31 (1.98–2.69)	< .001	2.33 (1.98–2.74)	< .001	0.725	0.882
HipC	2.7 (2.2–3.3)	< .001	3.27 (2.58–4.16)	< .001	0.739	0.859	2.01 (1.74–2.32)	< .001	2.07 (1.77–2.41)	< .001	0.694	0.881
Waist_Hip	2.89 (2.35–3.56)	< .001	3.11 (2.47–3.92)	< .001	0.752	0.857	2.43 (2.06–2.86)	< .001	2.38 (2.01–2.83)	< .001	0.727	0.872
Waist_Pelvic	2.34 (1.93–2.84)	< .001	2.24 (1.83–2.74)	< .001	0.712	0.849	1.88 (1.62–2.18)	< .001	1.89 (1.62–2.21)	< .001	0.674	0.863
Forehead_Waist	0.22 (0.17–0.28)	< .001	0.22 (0.16–0.29)	< .001	0.812	0.878	0.3 (0.25–0.36)	< .001	0.31 (0.25–0.37)	< .001	0.779	0.892
Forehead_Rib	0.25 (0.2–0.32)	< .001	0.25 (0.19–0.32)	< .001	0.791	0.867	0.32 (0.27–0.39)	< .001	0.33 (0.28–0.4)	< .001	0.776	0.887
Forehead_Chest	0.35 (0.28–0.43)	< .001	0.35 (0.28–0.44)	< .001	0.744	0.852	0.31 (0.26–0.37)	< .001	0.3 (0.25–0.37)	< .001	0.78	0.889
WHtR	4.38 (3.42–5.62)	< .001	4.56 (3.51–5.94)	< .001	0.818	0.888	3.1 (2.61–3.67)	< .001	3.05 (2.55–3.64)	< .001	0.79	0.897
TG	4.36 (3.26–5.83)	< .001	4.74 (3.47–6.46)	< .001	0.827	–	6.48 (5.07–8.3)	< .001	6.99 (5.4–9.06)	< .001	0.848	–
HW phenotype	0.02 (0.01–0.04)	< .001	0.02 (0.01–0.04)	< .001	–	–	0.05 (0.03–0.07)	< .001	0.05 (0.03–0.07)	< .001	–	–

The results were obtained by binary logistic regression. *M-40-49* men aged 40–49 years, *W-40-49* women aged 40–49 years, *Adj. p and OR* adjustment for age, region, and education, *OR* odds ratio, *AUC-1* AUC value of each index used to identify MetS, *AUC-2* AUC value of phenotypes combining TG + one anthropometric index to identify MetS

**Table 3:** Analysis of the association between MetS and anthropometric indices and the predictive power for MetS in men and women aged 50–59 years

M-50-59	M-50-59						W-50-59					
	OR	*p*	Adj. OR	Adj. p	AUC-1	AUC-2	OR	*p*	Adj. OR	Adj. p	AUC-1	AUC-2
Height	1.16 (1.05–1.28)	.003	1.16 (1.04–1.28)	.006	0.542	0.793	0.92 (0.85–1.01)	.066	0.96 (0.88–1.05)	.413	0.517	0.833
Weight	2.51 (2.23–2.83)	< .001	2.68 (2.37–3.04)	< .001	0.743	0.832	1.97 (1.8–2.17)	< .001	1.93 (1.75–2.13)	< .001	0.686	0.858
BMI	2.67 (2.35–3.02)	< .001	2.84 (2.49–3.23)	< .001	0.745	0.83	2.15 (1.95–2.37)	< .001	2.12 (1.91–2.34)	< .001	0.71	0.861
ForeheadC	1.46 (1.32–1.63)	< .001	1.51 (1.35–1.69)	< .001	0.612	0.797	1.21 (1.12–1.32)	< .001	1.24 (1.13–1.37)	< .001	0.558	0.834
NeckC	2.71 (2.4–3.06)	< .001	2.74 (2.41–3.12)	< .001	0.745	0.836	2.31 (2.08–2.55)	< .001	2.28 (2.05–2.54)	< .001	0.71	0.866
AxillaryC	2.57 (2.27–2.9)	< .001	2.5 (2.21–2.83)	< .001	0.739	0.833	2.49 (2.24–2.76)	< .001	2.39 (2.14–2.66)	< .001	0.735	0.872
ChestC	2.69 (2.38–3.05)	< .001	2.62 (2.31–3)	< .001	0.751	0.839	2.61 (2.35–2.91)	< .001	2.49 (2.23–2.78)	< .001	0.746	0.874
RibC	3.18 (2.79–3.63)	< .001	3.13 (2.73–3.58)	< .001	0.782	0.849	2.66 (2.39–2.96)	< .001	2.52 (2.25–2.81)	< .001	0.756	0.874
WaistC	3.54 (3.08–4.08)	< .001	3.46 (3–3.99)	< .001	0.805	0.868	2.54 (2.29–2.82)	< .001	2.41 (2.16–2.69)	< .001	0.745	0.877
PelvicC	2.46 (2.18–2.78)	< .001	2.57 (2.26–2.91)	< .001	0.736	0.836	2.03 (1.84–2.23)	< .001	1.93 (1.75–2.13)	< .001	0.694	0.861
HipC	2.21 (1.97–2.48)	< .001	2.29 (2.023–2.58)	< .001	0.71	0.828	1.67 (1.52–1.82)	< .001	1.62 (1.48–1.78)	< .001	0.639	0.851
Waist_Hip	2.52 (2.23–2.86)	< .001	2.54 (2.23–2.89)	< .001	0.731	0.826	2.44 (2.19–2.7)	< .001	2.34 (2.09–2.61)	< .001	0.726	0.868
Waist_Pelvic	2.24 (1.99–2.51)	< .001	2.11 (1.88–2.38)	< .001	0.714	0.818	1.92 (1.75–2.12)	< .001	1.8 (1.63–1.99)	< .001	0.674	0.857
Forehead_Waist	0.29 (0.25–0.33)	< .001	0.29 (0.25–0.34)	< .001	0.78	0.852	0.38 (0.34–0.42)	< .001	0.4 (0.36–0.45)	< .001	0.736	0.876
Forehead_Rib	0.34 (0.29–0.38)	< .001	0.34 (0.3–0.39)	< .001	0.753	0.831	0.37 (0.33–0.41)	< .001	0.4 (0.35–0.44)	< .001	0.747	0.873
Forehead_Chest	0.41 (0.37–0.47)	< .001	0.43 (0.38–0.48)	< .001	0.717	0.821	0.39 (0.35–0.43)	< .001	0.41 (0.36–0.46)	< .001	0.734	0.871
WHtR	3.32 (2.89–3.81)	< .001	3.26 (2.83–3.75)	< .001	0.791	0.856	2.56 (2.3–2.84)	< .001	2.44 (2.18–2.73)	< .001	0.746	0.874
TG	3.52 (2.98–4.17)	< .001	3.67 (3.09–4.37)	< .001	0.792	–	5.79 (4.95–6.77)	< .001	6.34 (5.37–7.48)	< .001	0.834	–
HW phenotype	0.04 (0.02–0.05)	< .001	0.03 (0.02–0.05)	< .001	–	–	0.07 (0.05–0.08)	< .001	0.07 (0.05–0.09)	< .001	–	–

The results were obtained by binary logistic regression. *M-50-59* men aged 50–59 years, *W-50-59* women aged 50–59 years, *Adj. p and OR* adjustment for age, region, and education, *OR* odds ratio, *AUC-1* AUC value of each index used to identify MetS, *AUC-2* AUC value of phenotypes combining TG + one anthropometric index to identify MetS

**Table 4:** Analysis of the association between MetS and anthropometric indices and the predictive power for MetS in men and women aged 60–69 years

Index	M-60-69						W-60-69					
	OR	*p*	Adj. OR	Adj. p	AUC-1	AUC-2	OR	*p*	Adj. OR	Adj. p	AUC-1	AUC-2
Height	1.18 (1.04–1.34)	.013	1.17 (1.02–1.34)	.029	0.538	0.788	1.03 (0.93–1.14)	.608	1.1 (0.98–1.23)	.101	0.473	0.79
Weight	2.4 (2.05–2.81)	< .001	2.71 (2.28–3.2)	< .001	0.729	0.826	1.67 (1.49–1.88)	< .001	1.79 (1.58–2.02)	< .001	0.638	0.805
BMI	2.58 (2.19–3.04)	< .001	2.95 (2.46–3.52)	< .001	0.74	0.833	1.73 (1.54–1.94)	< .001	1.8 (1.6–2.04)	< .001	0.648	0.808
ForeheadC	1.599 (1.38–1.82)	< .001	1.65 (1.42–1.91)	< .001	0.615	0.798	1.24 (1.11–1.37)	< .001	1.22 (1.08–1.36)	< .001	0.546	0.798
NeckC	2.56 (2.17–3)	< .001	2.57 (2.16–3.06)	< .001	0.73	0.827	1.97 (1.75–2.23)	< .001	1.87 (1.65–2.12)	< .001	0.672	0.821
AxillaryC	2.63 (2.23–3.1)	< .001	2.62 (2.2–3.11)	< .001	0.741	0.838	2.09 (1.85–2.36)	< .001	1.98 (1.74–2.25)	< .001	0.692	0.824
ChestC	2.84 (2.4–3.36)	< .001	2.87 (2.4–3.43)	< .001	0.755	0.845	2.17 (1.92–2.46)	< .001	2.06 (1.81–2.35)	< .001	0.704	0.826
RibC	3.34 (2.79–4)	< .001	3.38 (2.79–4.09)	< .001	0.777	0.852	2.18 (1.92–2.47)	< .001	2.05 (1.8–2.33)	< .001	0.704	0.825
WaistC	3.78 (3.11–4.59)	< .001	3.88 (3.17–4.76)	< .001	0.805	0.864	2.3 (2.02–2.61)	< .001	2.23 (1.96–2.55)	< .001	0.71	0.831
PelvicC	2.28 (1.95–2.65)	< .001	2.43 (2.06–2.86)	< .001	0.713	0.819	1.84 (1.63–2.07)	< .001	1.81 (1.6–2.05)	< .001	0.664	0.816
HipC	2.21 (1.9–2.58)	< .001	2.25 (1.92–2.65)	< .001	0.711	0.821	1.58 (1.41–1.77)	< .001	1.55 (1.38–1.75)	< .001	0.622	0.809
Waist_Hip	2.81 (2.37–3.34)	< .001	2.97 (2.48–3.57)	< .001	0.744	0.832	2.09 (1.85–2.36)	< .001	2.09 (1.83–2.38)	< .001	0.686	0.82
Waist_Pelvic	2.51 (2.14–2.95)	< .001	2.44 (2.06–2.9)	< .001	0.729	0.826	1.79 (1.59–2.01)	< .001	1.73 (1.53–1.96)	< .001	0.651	0.811
Forehead_Waist	0.27 (0.22–0.33)	< .001	0.27 (0.21–0.33)	< .001	0.779	0.849	0.45 (0.39–0.51)	< .001	0.45 (0.39–0.51)	< .001	0.695	0.827
Forehead_Rib	0.35 (0.29–0.42)	< .001	0.35 (0.29–0.42)	< .001	0.741	0.831	0.48 (0.42–0.54)	< .001	0.5 (0.44–0.57)	< .001	0.689	0.821
Forehead_Chest	0.42 (0.36–0.5)	< .001	0.43 (0.36–0.51)	< .001	0.71	0.823	0.48 (0.43–0.55)	< .001	0.5 (0.44–0.57)	< .001	0.686	0.821
WHtR	3.5 (2.9–4.22)	< .001	3.7 (3.03–4.53)	< .001	0.789	0.859	2.17 (1.92–2.46)	< .001	2.13 (1.87–2.43)	< .001	0.699	0.829
TG	2.98 (2.49–3.55)	< .001	3.05 (2.53–3.66)	< .001	0.788	–	5.71 (4.58–7.13)	< .001	6.08 (4.81–7.68)	< .001	0.792	–
HW phenotype	0.02 (0.01–0.04)	< .001	0.02 (0.01–0.04)	< .001	–	–	0.11 (0.09–0.15)	< .001	0.11 (0.08–0.14)	< .001	–	–

The results were obtained by binary logistic regression. *M-60-69* men aged 60–69 years, *W-60-69* women aged 60–69 years, *Adj. p and OR* adjustment for age, region, and education, *OR* odds ratio, *AUC-1* AUC value of each index used to identify MetS, *AUC-2* AUC value of phenotypes combining TG + one anthropometric index to identify MetS

**Table 5:** Analysis of the association between MetS and anthropometric indices and the predictive power for MetS in men and women aged 70–79 years

Index	M-70-79						W-70-79					
	OR	*p*	Adj. OR	Adj. p	AUC-1	AUC-2	OR	*p*	Adj. OR	Adj. p	AUC-1	AUC-2
Height	1.3 (1.09–1.54)	.003	1.28 (1.07–1.53)	.007	0.549	0.805	1.33 (1.16–1.51)	< .001	1.33 (1.16–1.53)	< .001	0.57	0.797
Weight	2.6 (2.11–3.21)	< .001	2.89 (2.3–3.65)	< .001	0.741	0.848	1.96 (1.69–2.27)	< .001	2.14 (1.82–2.51)	< .001	0.675	0.821
BMI	2.6 (2.09–3.21)	< .001	2.83 (2.25–3.56)	< .001	0.74	0.845	1.79 (1.55–2.06)	< .001	1.93 (1.66–2.25)	< .001	0.652	0.818
ForeheadC	1.47 (1.25–1.75)	< .001	1.46 (1.21–1.75)	< .001	0.602	0.811	1.27 (1.11–1.45)	< .001	1.25 (1.08–1.43)	0.002	0.559	0.797
NeckC	2.38 (1.95–2.91)	< .001	2.35 (1.9–2.9)	< .001	0.717	0.843	1.94 (1.68–2.26)	< .001	1.91 (1.64–2.23)	< .001	0.67	0.816
AxillaryC	2.22 (1.83–2.7)	< .001	2.28 (1.85–2.81)	< .001	0.709	0.838	1.9 (1.65–2.2)	< .001	1.94 (1.67–2.26)	< .001	0.67	0.821
ChestC	2.61 (2.12–3.21)	< .001	2.68 (2.15–3.33)	< .001	0.747	0.857	2.1 (1.78–2.39)	< .001	2.11 (1.81–2.47)	< .001	0.688	0.829
RibC	3.15 (2.51–3.96)	< .001	3.15 (2.49–3.99)	< .001	0.776	0.863	2.02 (1.74–2.4)	< .001	2 (1.71–2.34)	< .001	0.683	0.825
WaistC	3.8 (2.96–4.89)	< .001	3.87 (2.99–5.02)	< .001	0.804	0.876	2.12 (1.82–2.47)	< .001	2.15 (1.84–2.51)	< .001	0.687	0.831
PelvicC	2.84 (2.27–3.56)	< .001	2.98 (2.35–3.77)	< .001	0.748	0.853	1.85 (1.6–2.14)	< .001	1.9 (1.63–2.22)	< .001	0.659	0.824
HipC	2.58 (2.09–3.17)	< .001	2.63 (2.11–3.27)	< .001	0.734	0.856	1.72 (1.49–1.98)	< .001	1.74 (1.49–2.03)	< .001	0.642	0.818
Waist_Hip	2.55 (2.07–3.14)	< .001	2.63 (2.11–3.27)	< .001	0.732	0.843	1.8 (1.56–2.08)	< .001	1.89 (1.62–2.19)	< .001	0.646	0.816
Waist_Pelvic	2.22 (1.82–2.7)	< .001	2.25 (1.84–2.77)	< .001	0.71	0.842	1.67 (1.45–1.92)	< .001	1.68 (1.45–1.94)	< .001	0.631	0.811
Forehead_Waist	0.24 (0.19–0.32)	< .001	0.24 (0.18–0.32)	< .001	0.792	0.871	0.46 (0.39–0.54)	< .001	0.44 (0.38–0.52)	< .001	0.679	0.833
Forehead_Rib	0.33 (0.26–0.41)	< .001	0.32 (0.25–0.41)	< .001	0.759	0.856	0.5 (0.43–0.58)	< .001	0.5 (0.43–0.58)	< .001	0.674	0.824
Forehead_Chest	0.43 (0.35–0.52)	< .001	0.41 (0.33–0.5)	< .001	0.717	0.844	0.49 (0.43–0.57)	< .001	0.47 (0.41–0.55)	< .001	0.679	0.828
WHtR	3.26 (2.58–4.13)	< .001	3.36 (2.63–4.29)	< .001	0.778	0.863	1.84 (1.59–2.12)	< .001	1.87 (1.61–2.17)	< .001	0.656	0.822
TG	3.84 (2.97–5)	< .001	4.08 (3.11–5.36)	< .001	0.8	–	7.33 (5.43–9.91)	< .001	7.55 (5.52–10.32)	< .001	0.793	–
HW phenotype	0.03 (0.02–0.07)	< .001	0.03 (0.01–0.07)	< .001	–	–	0.08 (0.05–0.11)	< .001	0.08 (0.05–0.11)	< .001	–	–

The results were obtained by binary logistic regression. *M-70-79* men aged 70–79 years, *W-70-79* women aged 70–79 years, *Adj. p and OR* adjustment for age, region, and education, *OR* odds ratio, *AUC-1* AUC value of each index used to identify MetS, *AUC-2* AUC value of phenotypes combining TG + one anthropometric index to identify MetS

In men, WaistC and Forehead_Waist were the individual anthropometric indices most strongly associated with MetS in M-30-39 (odds ratio (OR) = 3.6 [95% CI, 2.59–5], adjusted OR = 3.85 [2.69–5.5] and OR = 0.28 [0.2–0.39], adjusted OR = 0.26 [0.18–0.37], respectively), M-40-49 (OR = 4.66 [3.6–6.02], adjusted OR = 4.95 [3.75–6.52] and OR = 0.22 [0.17–0.28], adjusted OR = 0.22 [0.16–0.29], respectively), and M-50-50 (OR = 3.54 [3.08–4.08], adjusted OR = 3.46 [3–3.99] and OR = 0.29 [0.25–0.33], adjusted OR = 0.29 [0.25–0.34], respectively). These indices remained the most strongly associated with MetS after adjusting for age, region, and education. In the M-60-69 group, WaistC displayed the strongest association with MetS (OR = 3.78 [3.11–4.59], adjusted OR = 3.88 [3.17–4.76]), and Forehead_Waist had the strongest association with MetS in the M-70-79 group (OR = 0.24 [0.19–0.32], adjusted OR = 0.24 [0.18–0.32]).

For women, Forehead_Waist showed the strongest associations with MetS in the W-30-39 group (OR = 0.21 [0.15–0.29], adjusted OR = 0.18 [0.12–0.27]) and W-40-49 group (OR = 0.3 [0.25–0.36], adjusted OR = 0.31 [0.25–0.37]). Unexpectedly, Forehead_Rib and RibC had the strongest association with MetS in the W-50-59 group (OR = 0.37 [0.33–0.41], adjusted OR = 0.4 [0.35–0.44] and OR = 2.66 [2.39–2.96], adjusted OR = 2.52 [2.25–2.81], respectively). In the W-60-69 group, WaistC and Forehead_Waist had the highest association with MetS (OR = 2.3 [2.02–2.61], adjusted OR = 2.23 [1.96–2.55] and OR = 0.45 [0.39–0.51], adjusted OR = 0.45 [0.39–0.51], respectively). Additionally, in the W-70-79 group, WaistC and Forehead_Waist were the most strongly associated with MetS (OR = 2.12 [1.82–2.47], adjusted OR = 2.15 [1.84–2.51] and OR = 0.46 [0.39–0.54], adjusted OR = 0.44 [0.38–0.52], respectively).

### Association of MetS with the HW phenotype and its components

The HW phenotype had the strongest association with MetS among all variables used in this study (Tables 1, 2, 3, 4 and 5). The HW phenotype was the variable most strongly associated with MetS in the M-30-39 group (OR = 0.02 [0.01–0.04], adjusted OR = 0.02 [0.01–0.04]) and the W-30-39 group (OR = 0.01 [0.01–0.03], adjusted OR = 0.01 [0.01–0.02]). The strength of the association between the HW phenotype and MetS was higher in men than in women, with the exception of the W-30-39 group. The HW phenotype showed the lowest association with MetS in the W-60-69 group of all age and gender groups (OR = 0.11 [0.09–0.15], adjusted OR = 0.11 [0.08–0.14]).

The HW phenotype consists of TG and WaistC. In men, the strength of the associations between MetS and TG and between MetS and WaistC changed frequently according to age group; this trend differed from that in women. Specifically, when comparing WaistC and TG as components of the HW phenotype in M-30-39, TG (OR = 5.82 [3.76–8.99], adjusted OR = 5.68 [3.63–8.89]) was more strongly associated with MetS than WaistC (OR = 3.6 [2.59–5], adjusted OR = 3.85 [2.69–5.5]). However, MetS showed stronger associations with WaistC than with TG in both M-40-49 (OR = 4.66 [3.6–6.02], adjusted OR = 4.95 [3.75–6.52] in WaistC and OR = 4.36 [3.26–5.83], adjusted OR = 4.74 [3.47–6.46] in TG) and M-60-69 (OR = 3.78 [3.11–4.59], adjusted OR = 3.88 [3.17–4.76] in WaistC and OR = 2.98 [2.49–3.55], adjusted OR = 3.05 [2.53–3.66] in TG). In the M-50-59 group, the associations between WaistC and MetS (OR = 3.54 [3.08–4.08], adjusted OR = 3.46 [3–3.99]) and between TG and MetS (OR = 3.52 [2.98–4.17], adjusted OR = 3.67 [3.09–4.37]) were similar. In the M-70-79 group, MetS was more strongly associated with TG (OR = 3.84 [2.97–5], adjusted OR = 4.08 [3.11–5.36]) than WaistC (OR = 3.8 [2.96–4.89], adjusted OR = 3.87 [2.99–5.02]). TG showed stronger associations with MetS than did WaistC in all groups of women except the W-30-39 group.

### Analysis of the ability of individual anthropometric indices and various phenotypes to identify MetS

In the analysis of the predictive power of individual anthropometric indices (AUC-1 in Tables 1, 2, 3, 4 and 5), WaistC was best indicator of MetS in men of all age groups. However, in women, WaistC was the strongest indicator of MetS in only the W-30-39 (AUC = 0.883) and W-60-69 groups (AUC = 0.71). In the W-40-49 group, WHtR was the best indicator of MetS (AUC = 0.79). Additionally, RibC and ChestC were the strongest indicators in the W-50-59 (AUC = 0.756) and W-70-79 groups (AUC = 0.688).

Regarding the predictive ability of combinations of TG with individual anthropometric indices (AUC-2 in Tables 1, 2, 3, 4, 5), the combination of WaistC and TG was the best indicator of MetS in men and women overall. However, interestingly, TG + WHtR was the best indicator of MetS in W-40-49 (AUC = 0.897). Additionally, in the W-30-39 group and the M-40-49 group, TG + WHtR had a similar predictive ability to that of the best indicator in these groups. TG + WaistC (AUC = 0.876) and TG + Forehead_Waist (AUC = 0.871) in M-70-79 were the best indicators of MetS, and TG + Forehead_Waist was the best indictor in W-70-79 (AUC = 0.833). The predictive power of TG with WaistC was the highest in the youngest groups in this study (M-30-39 and W-30-39 groups) compared with the other older groups. When identifying MetS using actual TG values combined with single anthropometric index values, adding TG to the single index in women highly improved the predictive power compared to that of the single measurement in all age groups except for the W-30-39 group.

## Discussion

Using various anthropometric indices is critical to identifying several obesity-related chronic diseases because the anthropometric index that best predicts a particular chronic disease such as MetS, type 2 diabetes, hypertension, hypotension, hypertriglyceridemia, dyslipidemia, or cardiovascular disease depends on and may differ by each condition [7, 11, 25, 26, 37–41].

Many studies to date have examined the association of MetS with several anthropometric indices such as BMI, WaistC, WHtR, and WHR to determine the best indicator of MetS in different countries [7–13, 42–45]. However, the best anthropometric indicator of MetS remains controversial, even though WaistC is one of the five components used to diagnose MetS and is a strong predictor of MetS. On the one hand, Gharipour and colleagues [7] have commented that WaistC is better than BMI and WHR in diagnosing MetS among Iranian adults, regardless of age and gender. Moreno and colleagues [42] tried to identify the best anthropometric indicator of MetS in non-obese children and children with exogenous obesity. They found that the useful indicators of MetS in children were BMI, WaistC, and triceps/subscapular skinfold ratio and suggested that WaistC seemed to be the best indicator of MetS in children. Wang and colleagues [8] compared the predictive power of WaistC, BMI, and WHR to identify MetS in Chinese adults, and they argued that BMI and WaistC had higher predictive value than WHR. Additionally, Bener and colleagues [9] reported that compared with BMI, WHR, and WHtR, WaistC with cut-off values of 91 cm in women and 99.5 cm in men was the best indicator of MetS in a Qatari adult population. Choi and colleagues [43] documented that WaistC, BMI, and WHtR had similar predictive power for MetS in a Korean population.

On the other hand, several studies have compared individual anthropometric indices and suggested that the best index for MetS diagnosis is not WaistC [10–13, 44, 45]. Hsieh and Muto [11] assessed the association of MetS risk factors with BMI, WaistC, and WHtR in Japanese men and women and reported that WHtR was the best indicator for MetS; specifically, WHtR ≥0.5 was suggested to be the most effective index for screening Japanese adults. Shao and colleagues [12] examined the relationship of MetS risk factors with BMI, WaistC, WHR, and WHtR in Chinese adults and found that WHtR > 0.5 was the strongest indicator of MetS risk factors. Mombelli and colleagues [13] tested several anthropometric indices for MetS diagnoses in a non-obese Italian population and reported that the strongest index for screening high-risk patients for MetS was WHtR, not WaistC or BMI. Elizondo-Montemayor and colleagues [10] identified the usefulness of WaistC, BMI, and WHtR for the prediction of MetS in Mexican children. They suggested that WHtR > 0.59 was a strong indicator of MetS in a Mexican population. Additionally, Rodea-Montero and colleagues [44] reported that WHtR was a better indicator of MetS in obese Mexican adolescents than BMI and WaistC. Most of our findings are consistent with the results of previous studies [6–9, 42] indicating that WaistC was the best indicator of MetS; however, interestingly, we found that the best indicators were WHtR in women aged 40–49 years, RibC in women aged 50–59 years, and ChestC in women aged 70–79 years.

Many studies to date have suggested that the HW phenotype can be applied as an alternative and important indicator of MetS because it can be measured using a simple and cost-effective method. Gomez-Huelgas and colleagues [14] examined the association of the HW phenotype with MetS, type 2 diabetes, and cardiovascular disease in an adult Spanish population. They argued that the prevalence of HW phenotype was significantly higher in men than in women and that the HW phenotype might be an alternative to MetS criteria for predicting diabetes and cardiovascular disease. Rosolova and colleagues [19] reported that the HW phenotype predicted MetS and was a strong indicator of coronary risk in subjects with type 2 diabetes, regardless of age and gender. Additionally, Lee and colleagues [15] examined the associations of HW phenotype with metabolic abnormalities such as blood pressure, TG, total cholesterol, and HDL and LDL cholesterol in Korean women and showed that the HW phenotype was a useful indicator of metabolic abnormalities. Esmaillzadeh and colleagues [20] documented that the HW phenotype was a simple indicator of MetS and metabolic abnormalities in Iranian adolescents. Nawabzad and Champin [21] assessed the concordance between three criteria including the HW phenotype, the International Diabetes Federation (IDF) criteria, and the NCEP ATP III criteria for MetS in the French population and obtained Kappa concordance coefficients of 0.46 between the NCEP ATP III and HW phenotype and 0.43 between HW phenotype and the IDF criteria. The authors argued that compared with the IDF and NCEP ATP III criteria, the HW phenotype was a useful tool for identifying MetS. Lemieux and colleagues [22] tested the usefulness of the HW phenotype for screening for metabolic risks of CHD in men from the Québec City metropolitan area and Saguenay–Lac-St-Jean regional hospital in Chicoutimi and reported that the HW phenotype could be a useful tool for predicting atherogenic MetS because of the simple measurement of the phenotype, the inexpensive screening, and the simple interpretation of WaistC and fasting TG. Our findings were consistent with the results of previous studies [14, 15, 19, 20], indicating that the HW phenotype was a useful tool for MetS. However, although they combined TG and WaistC as components of the HW phenotype, these previous studies did not examine alternative circumference or ratio indices that could replace WaistC in the HW phenotype to identify MetS. We found that the phenotype with the highest predictive power for MetS was the combinations of TG + WHtR in the W-40-49 group and TG + Forehead_Waist in the W-70-79 group, indicating the utility of two different components of the HW phenotype. Although the predictive power of the combination of TG and WaistC for MetS was high in most age and gender groups compared with that of other phenotypes, our findings indicated that the best phenotype (combination) of MetS may differ according to age group, especially in women.

Several limitations of the present study should be noted. First, the main limitation is that cause-effect relationships cannot be determined due to the cross-sectional nature of the study design. Second, we used the NCEP ATP III criteria to identify MetS. In the NCEP ATP III, the criterion for abdominal obesity is WaistC > 102 cm in men and > 88 cm in women; however, these values may not be suitable for Asian populations. Therefore, a WaistC > 80 cm in women and > 90 cm in men was used in this study because we believed that that criterion was a more appropriate definition of central obesity in this population.

## Conclusion

In the present study, we identified the best indicator of MetS among various anthropometric indices and examined the power of various phenotypes that combined individual anthropometric indices with TG levels to identify MetS. We found that in middle-aged men and elderly women, WaistC and Forehead_Waist showed the strongest association with MetS. Regarding the HW phenotype, the strength of the associations between MetS and TG and between MetS and WaistC changed frequently according to age group in men; this finding differed from the trend in women. Additionally, when comparing single anthropometric indexes with the combination of one index and TG, the improvement in predictive power using the combination of an index and TG was particularly higher in women than in men, except for in the W-30-39 group. In women, but not in men, the best indicator of MetS among the individual anthropometric indices and the various phenotypes combining a single anthropometric index and TG may differ subtly according to age group. Our findings provide insight into a simple and inexpensive method that could be used to identify MetS in initial health screening efforts in a Korean population.

## Supplementary information

**Additional file 1: Supplementary Table 1.** Basic characteristics and brief descriptions of variables used in this study. **Supplementary Table 2.** Baseline data between normal and MetS groups in men and women.

## Data Availability

Data are available from the Korean Health and Genome Epidemiology Study (KHGES) database Institutional Data Access / Ethics Committee and the Korea Institute of Oriental Medicine (KIOM) Korean medicine data center (KDC, http://kdc.kiom.re.kr/html/, permission number: 20130903–20140327, Bum Ju Lee) for researchers who meet the criteria for access to confidential data. The criteria for data access are the rationality of research topics or hypotheses, the rationality of research methods, and compliance with research ethics regulations.

## Abbreviations

AUC: Area under the receiver operating characteristic curve
BMI: Body mass index
Forehead_Rib: Forehead-to-rib ratio
Forehead_Waist: Forehead-to-waist ratio
HW: Hypertriglyceridemic waist
MetS: Metabolic syndrome
TG: Triglyceride
WC and WaistC: Waist circumference
WHR: Waist-to-hip ratio
WHtR: Waist-to-height ratio
