# Supplementary material for: Identification of metabolic syndrome using phenotypes consisting of triglyceride levels with anthropometric indices in Korean adults
Source: BMC Endocr Disord. 2020 Feb 27;20:29. doi: 10.1186/s12902-020-0510-0 (PMC7045372; doi:10.1186/s12902-020-0510-0)
Supplement: Supplementary file 1 — Additional file 1: Supplementary Table 1. Basic characteristics and brief descriptions of variables used in this study. Supplementary Table 2. Baseline data between normal and MetS groups in men and women. [file 12902_2020_510_MOESM1_ESM.docx]

Supplementary table 1. Basic characteristics and brief descriptions of variables used in this study.

| Variable | Men | Women |
| --- | --- | --- |
| Subjects | 4936 | 7089 |
| Height (cm) ^‡^ | 168 (6.06) | 155 (6.01) |
| Weight (kg)^‡^ | 68.6 (10.1) | 58.1 (8.35) |
| BMI ^‡^ | 24.4 (2.99) | 24.1 (3.29) |
| Pulse ^‡^ | 68.5 (9.54) | 69.4 (9.33) |
| Age ^‡^ | 56.2 (11) | 55.1 (11.7) |
| ForeheadC (cm) ^‡^ | 56.9 (1.79) | 55 (1.74) |
| NeckC (cm) ^‡^ | 37.8 (2.49) | 33.5 (2.21) |
| AxillaryC (cm) ^‡^ | 95.8 (6.09) | 88.2 (6.1) |
| ChestC (cm) ^‡^ | 94.1 (6.38) | 91 (7.88) |
| RibC (cm) ^‡^ | 88.2 (6.63) | 80.2 (8.04) |
| WaistC (cm) ^‡^ | 87.5 (8.03) | 84.5 (9.07) |
| PelvicC (cm) ^‡^ | 91.6 (6.27) | 90.9 (7.16) |
| HipC (cm) ^†^ | 93.4 (5.77) | 93.1 (5.98) |
| Waist_Hip ^‡^ | 0.94 (0.06) | 0.91 (0.07) |
| Waist_Pelvic ^‡^ | 0.96 (0.05) | 0.93 (0.06) |
| Forehead_Waist ^*^ | 0.65 (0.06) | 0.66 (0.07) |
| Forehead_Rib ^‡^ | 0.65 (0.05) | 0.69 (0.07) |
| Forehead_Chest ^†^ | 0.61 (0.04) | 0.61 (0.05) |
| WHtR ^‡^ | 0.52 (0.05) | 0.55 (0.06) |
| AST (IU/L) ^‡^ | 27.9 (17) | 24 (10.8) |
| ALT (IU/L) ^‡^ | 27.9 (17.7) | 21.7 (14.4) |
| BUN (mg/dL) ^‡^ | 15.9 (4.5) | 14.6 (4.21) |
| Creatinine (mg/dL) ^‡^ | 1.05 (0.19) | 0.84 (0.17) |
| Glucose (mg/dL) ^‡^ | 103 (26.3) | 97.4 (24.8) |
| Total cholesterol (mg/dL) ^‡^ | 187 (34.5) | 194 (34.8) |
| Triglyceride (mg/dL) ^‡^ | 154 (112) | 125 (74.2) |
| HDL Cholesterol (mg/dL) ^‡^ | 44.1 (11.6) | 50.1 (13.3) |
| LDL Cholesterol (mg/dL) ^‡^ | 114 (32.5) | 117 (32.2) |
| Hemoglobin (g/dL) ^‡^ | 14.9 (1.22) | 12.9 (1.12) |
| Hematocrit (%) ^‡^ | 44.1 (3.63) | 38.2 (3.25) |
| Systolic BP (mmHg) ^‡^ | 122 (15.6) | 119 (16.9) |
| Diastolic BP (mmHg) ^‡^ | 80.4 (10.5) | 77 (10.7) |

The results were obtained by Student’s unpaired t-test. * p <0.05, † p <0.005, ‡ p <0.0001 indicate a significant difference between the men and women. Continuous variables are summarized as the mean (standard deviation). ForeheadC, forehead circumference; NeckC, neck circumference; AxillaryC, axillary circumference; ChestC, chest circumference; RibC, rib circumference; WaistC, waist circumference; PelvicC, pelvic circumference; HipC, hip circumference; Waist_Hip, waist-to-hip circumference ratio; Waist_Pelvi, waist-to-pelvic circumference ratio; Forehead_Waist, forehead-to-waist circumference ratio; Forehead_Rib, forehead-to-rib circumference ratio; Forehead_Chest, forehead-to-chest circumference ratio; WHtR, waist-to-height ratio; AST, aspartate aminotransferase; ALT, alanine aminotransferase; BUN, blood urea nitrogen; HDL, high-density lipoprotein; LDL, low-density lipoprotein; BP, blood pressure.

Supplementary table 2. Baseline data between normal and MetS groups in men and women.

| Variable | Men |  | Women |  |
| --- | --- | --- | --- | --- |
|  | Normal | MetS | Normal | MetS |
| Subjects | 4114 | 1465 | 5520 | 2365 |
| Height | 168 (6.91)^*^ | 168 (6.1) | 157 (6.27) ^‡^ | 154 (5.89) |
| Weight | 66.4 (10.1) ^‡^ | 74.6 (10.2) | 56.2 (8.14) ^‡^ | 61.2 (8.61) |
| BMI | 23.5 (2.92) ^‡^ | 26.2 (2.79) | 22.9 (3.18) ^‡^ | 25.8 (3.1) |
| Pulse | 68.5 (9.67) ^‡^ | 70.4 (9.87) | 69.5 (9.41) ^‡^ | 70.9 (9.68) |
| Age | 51.3 (16) ^‡^ | 55.6 (12.3) | 48.5 (15) ^‡^ | 60.1 (11.3) |
| ForeheadC | 56.8 (1.84) ^‡^ | 57.4 (1.89) | 55.1 (1.77) | 55.1 (1.77) |
| NeckC | 37.1 (2.41) ^‡^ | 39.4 (2.45) | 32.8 (2.11) ^‡^ | 34.5 (2.17) |
| AxillaryC | 94 (6.39) ^‡^ | 99.4 (5.82) | 86.1 (6.05) ^‡^ | 91.5 (5.6) |
| ChestC | 91.9 (6.71) ^‡^ | 98.3 (6.01) | 88 (7.66) ^‡^ | 95.6 (6.95) |
| RibC | 85.5 (6.84) ^‡^ | 92.9 (5.63) | 76.9 (7.69) ^‡^ | 85.2 (7.06) |
| WaistC | 84.5 (7.92) ^‡^ | 93.5 (6.73) | 80.9 (8.78) ^‡^ | 90.3 (7.63) |
| PelvicC | 89.7 (6.38) ^‡^ | 95.3 (5.93) | 88.5 (7.07) ^‡^ | 94.6 (6.57) |
| HipC | 92.3 (5.88) ^‡^ | 96.8 (5.8) | 91.9 (5.95) ^‡^ | 95.2 (6.14) |
| Waist_Hip | 0.92 (0.06) ^‡^ | 0.97 (0.05) | 0.88 (0.07) ^‡^ | 0.95 (0.06) |
| Waist_Pelvic | 0.94 (0.05) ^‡^ | 0.98 (0.04) | 0.91 (0.05) ^‡^ | 0.96 (0.05) |
| Forehead_Waist | 0.68 (0.06) ^‡^ | 0.62 (0.04) | 0.69 (0.07) ^‡^ | 0.61 (0.05) |
| Forehead_Rib | 0.67 (0.05) ^‡^ | 0.62 (0.04) | 0.72 (0.07) ^‡^ | 0.65 (0.05) |
| Forehead_Chest | 0.62 (0.04) ^‡^ | 0.59 (0.03) | 0.63 (0.05) ^‡^ | 0.58 (0.04) |
| WHtR | 0.5 (0.05) ^‡^ | 0.56 (0.04) | 0.52 (0.06) ^‡^ | 0.59 (0.05) |
| AST | 26.8 (16.5) ^‡^ | 29.9 (17.5) | 23.1 (10.4) ^‡^ | 24.8 (11.2) |
| ALT | 26 (18.5) ^‡^ | 32.6 (19.1) | 20 (13.4) ^‡^ | 24 (15.6) |
| BUN | 15.5 (4.26) | 15.7 (4.93) | 14 (4.06) ^‡^ | 14.9 (4.64) |
| Creatinine | 1.03 (0.16) ^‡^ | 1.08 (0.26) | 0.83 (0.14) ^‡^ | 0.87 (0.23) |
| Glucose | 96.5 (18.2) ^‡^ | 116 (35.2) | 91 (13.4) ^‡^ | 108 (35.7) |
| Total cholesterol | 183 (34.4) ^‡^ | 189 (36.4) | 188 (34.4) ^‡^ | 197 (35.6) |
| Triglyceride | 122 (73.7) ^‡^ | 224 (150) | 94.8 (40.9) ^‡^ | 179 (92.8) |
| HDL Cholesterol | 47.2 (11.6) ^‡^ | 37.5 (8.56) | 54.6 (13.1) ^‡^ | 41.4 (8.38) |
| LDL Cholesterol | 111 (31.2)^*^ | 114 (36.6) | 112 (31.6) ^‡^ | 122 (33.2) |
| Hemoglobin | 14.8 (1.21) ^‡^ | 15.1 (1.25) | 12.9 (1.1) ^‡^ | 13 (1.11) |
| HCT | 44 (3.59) ^‡^ | 44.7 (3.71) | 38.1 (3.17) ^‡^ | 38.5 (3.24) |
| Systolic BP | 119 (15) ^‡^ | 129 (14.9) | 114 (14.7) ^‡^ | 128 (16.6) |
| Diastolic BP | 78.1 (10.4) ^‡^ | 84.9 (10.3) | 74.1 (9.9) ^‡^ | 81.8 (10.7) |

The results were obtained by Student’s unpaired t-test. * p <0.05, † p <0.005, ‡ p <0.0001 indicate a significant difference between normal and MetS groups in men and women. Continuous variables are summarized as the mean (standard deviation).
